# Ultrastructural Characterization of Porcine Growing and In Vitro Matured Oocytes

**DOI:** 10.3390/ani10040664

**Published:** 2020-04-11

**Authors:** Michel Kere, Pan-Chen Liu, Yuh-Kun Chen, Pei-Chi Chao, Li-Kuang Tsai, Ting-Yu Yeh, Chawalit Siriboon, Payungsuk Intawicha, Neng-Wen Lo, Hsing-I Chiang, Yang-Kwang Fan, Jyh-Cherng Ju

**Affiliations:** 1 Department of Animal Science, National Chung Hsing University, 250 Kuo Kuang Road, Taichung 402, Taiwan; keremiche@yahoo.fr (M.K.); samchiang0127@gmail.com (H.-I.C.); ykfan@dragon.nchu.edu.tw (Y.-K.F.); 2 Institute of Rural Development, Nazi Boni University, 01 P.O. Box 1091 Bobo-Dioulasso 01, Burkina Faso; 3 Department of Veterinary Medicine, National Chung Hsing University, 250 Kuo Kuang Road, Taichung 402, Taiwan; pcliu@dragon.nchu.edu.tw; 4 Department of Plant Pathology, National Chung Hsing University, 250 Kuo Kuang Road, Taichung 402, Taiwan; ykchen@dragon.nchu.edu.tw (Y.-K.C.); pcchao@mail.nchu.edu.tw (P.-C.C.); 5 Bachelor Program of Biotechnology, National Chung Hsing University, No. 250, Kuokuang Rd., Taichung 402, Taiwan; leotsai123@hotmail.com; 6 Graduate Institute of Biotechnology, National Taiwan University, Taipei 10617, Taiwan; thurisaz0809@hotmail.com; 7 Department of Animal Science, Faculty of Agriculture, Ubon Ratchathani University, Ubon Ratchathani 34190, Thailand; csiriboon@hotmail.com; 8 Department of Animal Science, School of Agriculture and Natural Resources, University of Phayao, 19 Moo 2 Tambon Maeka Amphur Muang Phayao 56000, Thailand; payungsuk@hotmail.com; 9 Department of Animal Science and Biotechnology, Tunghai University, 181 Sec. 3 Taichung Harbor Road, Taichung 407, Taiwan; nlo@thu.edu.tw; 10 Graduate Institute of Biomedical Sciences, China Medical University, 91 Shueh Shih Rd., Taichung 40402, Taiwan; 11 Translational Medicine Center, China Medical University Hospital, 91 Shueh Shih Rd., Taichung 40402, Taiwan; 12 Department of Bioinformatics and Medical Engineering, Asia University, Taichung 41354, Taiwan

**Keywords:** oogenesis, organelles, mitochondria, lipid droplets, cortical granules

## Abstract

**Simple Summary:**

During oocyte growth and maturation, the organelle’s morphology of porcine oocytes changed and populated different compartments depending on the differentiation status. Changes in ultrastructural or subcellular level of porcine oocytes during oogenesis/folliculogenesis were observed, potentially leading to future mitochondrion replacement therapies of oocytes.

**Abstract:**

This study aimed to investigate ultrastructural changes of growing porcine oocytes and in vitro maturated oocytes. Light microscopy was used to characterize and localize the primordial, primary, secondary, and tertiary follicles. During oocyte growth and maturation, the morphology of mitochondria was roundish or ovoid in shape depending on the differentiation state, whereas their mean diameters oscillated between 0.5 and 0.7 µm, respectively, from primary and secondary follicles. Hooded mitochondria were found in the growing oocytes of the tertiary follicles. In addition to the pleomorphism of mitochondria, changes in the appearance of lipid droplets were also observed, along with the alignment of a single layer of cortical granules beneath the oolemma. In conclusion, our study is apparently the first report to portray morphological alterations of mitochondria that possess the hooded structure during the growth phase of porcine oocytes. The spatiotemporal and intrinsic changes during oogenesis/folliculogenesis are phenomena at the ultrastructural or subcellular level of porcine oocytes, highlighting an in-depth understanding of oocyte biology and impetus for future studies on practical mitochondrion replacement therapies for oocytes.

## 1. Introduction

The difficulty distinguishing developmental competent from incompetent eggs denotes our partial understanding of embryo quality-related characteristics and the timing for these specific characteristics being acquired during folliculo-ovogenesis. Organelles exert complicated associations within individual cells according to their energetic-metabolic needs during differentiation, maturation, and fertilization. These differential spatiotemporal capabilities associated with different organelles are inherited by the developing embryo, from which they eventually differentiate into more specific somatic cell lineages during later development [1,2,3,4]. During early embryogenesis, primordial germ cells (PGCs) migrate and colonize the genital ridges [5,6,7,8]. After that, PGCs enter meiosis and transform into oogonia, based on their chromosomal sex type, and then give rise to oocytes that are later surrounded by granulosa cells to form primordial follicles [6]. During folliculogenesis, morphological and functional alterations occur at the ultrastructural level in the oocyte nucleus (shape and position), mitochondrion (shape, number, and complex), lipid droplets, endoplasmic reticulum, Golgi complexes, zona pellucida, gap junctions, and annulate lamellae [9,10,11,12,13,14,15,16,17,18]. A better understanding of these ooplasmic events or characteristics would advance toward resolving the developmental obstacles in female infertility trough organelle’s transplantation.

To identify these changes, electron microscopy has been a powerful tool to reveal these events or aberrations within ultra-thin sections. Precise characterization at the cellular level during ovarian folliculo-ovogenesis is crucial for monitoring normal development of female gametes, as well as for improving the assisted reproductive techniques, such as oocyte cryopreservation and in vitro embryo production [3,4,19]. Therefore, the present study aimed to investigate the ultrastructural changes including mitochondrion size of porcine oocytes.

## 2. Materials and Methods 

### 2.1. Collection of Ovaries and Oocyte In Vitro Maturation (IVM)

Ovaries from prepubertal gilts (crossbred females) were collected at a local abattoir and transported to the laboratory in normal saline solution (0.9% NaCl) containing penicillin (600 IU/mL) at 37 °C within 1 h after slaughter as described in our previous studies [19]. In laboratory, ovaries were trimmed and rinsed with 70% ethanol and saline. Samples from ovarian cortex were taken for evaluation of various stages of folliculogenesis including preantral (primordial, primary, and secondary), antral follicles, and in vitro matured oocytes. Oocytes were aspirated from follicles (3–7 mm in diameter), and cumulus–oocyte complexes (COCs) possessing a homogeneous ooplasm were selected for maturation in North Carolina State University 23 (NCSU-23) medium. Then, 20–30 oocytes were randomly allocated to each 100-µL droplet of IVM medium covered by mineral oil and cultured at 39 °C in an incubator containing 5% CO_2_. For the first 22 h, COCs were cultured in NCSU-23 medium supplemented with 10% porcine follicular fluid, cysteine (0.1 mg/mL), equine chorionic gonadotrophin (10 IU/mL), and human chorionic gonadotrophin (10 IU/mL), and then the COCs from all treatment groups were switched to the medium without hormones for another 22 h.

### 2.2. Light Microscopic Evaluation

Minced pieces of the ovarian cortex were fixed in 10% formaldehyde (*V*/*V*) in 0.1 M phosphate-buffered saline (PBS, pH 7.2) at 4 °C overnight before the renewal of fixative solution for a second round. The fixed ovarian tissues were dehydrated in ethanol, clarified with xylene, and embedded in paraffin wax. Semi-serial sections (5 µm in thickness) were stained with hematoxylin and eosin (HE) and examined with a Zeiss Axiophot bright field light microscope (Zeiss, Oberkochen, Germany). Only morphologically normal follicles with visible nuclei were evaluated and images were captured with a digital CCD camera (Sony DXC-107A, Tokyo, Japan). Follicles were analyzed by light microscopy. The classification of follicle development observed in this study was based on Fair et al. [18] categorization for bovine follicles. Briefly, Fair et al. [20] divided them into five classes: (i) resting primordial follicles, with a single layer of flattened GCs; (ii) activated primordial follicles, with a single layer of both squamous and cuboidal GCs; (iii) primary follicles, with a single layer of cuboidal GCs; (iv) secondary follicles, with an incomplete or complete bilayer of cuboidal cells; and (v) early tertiary follicles, with more than two layers of GCs delineating one or several intercellular cavities.

### 2.3. Transmission Electron Microscopy (TEM)

All reagents for the electron microscopy were purchased from Electron Microscopy Sciences (Hatfield, PA, USA) and samples were processed as described in previous studies [20,21,22,23,24,25,26,27,28,29] with some modifications. Primordial, primary, secondary, antral follicles, and in vitro matured oocytes were evaluated by TEM. Portions of the ovarian cortex and dissected antral follicles were prefixed in 2% paraformaldehyde and 2% glutaraldehyde in 0.1 M PBS (pH 7.2) for 1 h. Once fixated, the tissue was washed by aspiration three times for 5 min each in fixation buffer. After checking the accurate follicle location, the tissue was further cut into small blocks (~1 mm^3^) for a second round of fixation at 4 °C overnight. Later, samples were post-fixed in solution containing 1.5% osmium tetroxide, 0.8% potassium ferricyanide, and 5 mM calcium chloride for 4 h, followed by dehydration in gradient acetone and then embedded in laboratory grade white resin (ProScitech, Kirwan, QLD, Australia). Ultra-thin sections (70 nm) were obtained from the samples mounted on copper grids (G200HF3-C, Gilder Grids, Lincolnshire, England), stained with 2% uranyl acetate (BDH Chemicals Ltd., Poole, England) for 30 min and then lead citrate (Sigma, St. Louis, Missouri, USA) for 10 min prior to examination with a transmission electron microscope (Jeol JEM 1400, Tokyo, Japan). Micrographs were taken, processed, and analyzed using Image J 1.46r (National Institute of Mental Health, Bethesda, MD, USA).

### 2.4. Morphometric Analysis of Mitochondria

Parameters applied in measurement were based on previous studies with minor modifications [1,17,30]. Briefly, measurements of mitochondrion diameter were performed using Image J 1.46r on micrographs from primordial follicles to matured oocytes. The numbers of mitochondria measured from each growing stage were: primordial follicle, 28; primary follicle, 28; secondary follicle, 33; tertiary follicle 29; and matured oocyte, 31. The numbers of oocytes used for mitochondria measurement out of those evaluated by TEM from each growing stage were: primordial follicle, 5/9; primary follicle, 5/11; secondary follicle, 4/7; tertiary follicle 4/11; and matured oocyte, 4/12. Data on the size of mitochondria were analyzed using ANOVA, and the Tukey’s test was used to detect differences among sample means by using commercial statistic package SPSS 17.0 (SPSS Inc., Chicago, IL, USA).

## 3. Results

### 3.1. Light Microscopic Structures Examined by HE Staining

**Figure 1:**
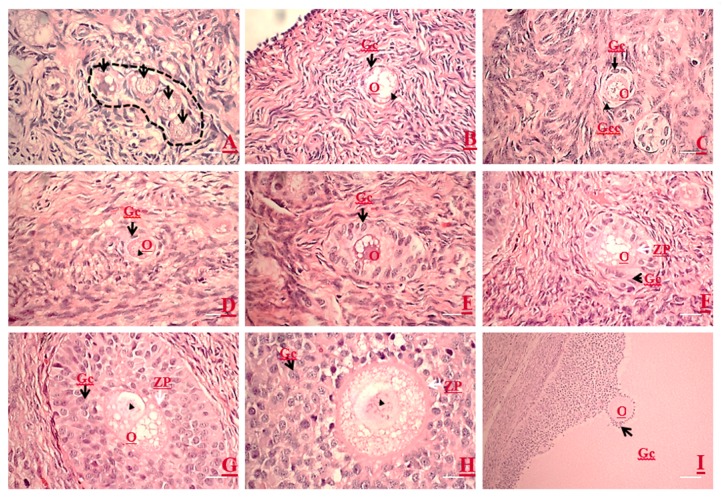
Microscopic morphologies of growing oocytes, follicles, and the associated follicular cells at different stages of folliculogenesis by using semi-thin sections of porcine ovaries. (**A**) A cluster of primordial follicles (enclosed in dot line) is observed in the cortex area. (**B**) Note the flattened granulosa cells (Gc) (arrow) are surrounding an immature oocyte, and the oocyte contains an eccentrically localized nucleus (arrowhead). (**C**) An activated primordial follicle: an oocyte is surrounded by two types of granulosa cells, i.e., cuboidal granulosa cells (arrow) at one pole and flattened granulosa cells (arrowhead). (**D**) A primary follicle oocyte is surrounded by a single layer of cuboidal granulosa cells (arrow), and the oocyte contains an eccentrically localized nucleus (arrowhead). (**E**–**H**) An early secondary follicle is transforming into a late stage secondary follicle with the onset of zona pellucida (ZP) formation (white arrow). Note that the increasing layers of cuboidal granulosa cells can be observed with no ZP structure (**E**), and all oocytes possess an eccentric germinal vesicle (GV nucleus, arrowhead). A very thin ZP (**F**) starts to form (white arrow). (**G**) The ZP is getting thicker as the granulosa layers increased. The granulosa cells start getting loosening while the ZP is thickening. (**I**) A tertiary follicle shows multiple layers of polar granulosa cells, antrum, and an eccentric cumulus–oocyte complex (COC). O, oocyte. Magnification: 100× (Scale bars, 100 µm) (**A**,**B**,**D**; **E**–**G**); 200× (scale bars, 50 µm) (**C**,**H**); and 40× (scale bar, 250 µm) (**I**).

In the primordial follicle, an oocyte is normally encompassed by a single layer of flattened granulosa cells. Primordial follicles are found in clusters and their oocyte exhibited ovoid to spherical in shape (Figure 1A), with a centered or eccentric voluminous nucleus (Figure 1B). Occasionally, cuboidal granulosa cells were present in some primordial follicles, which usually appeared at one pole of the follicle (Figure 1C). Primary follicles had one layer of cuboidal granulosa cells surrounding the oocyte in which the nucleus is voluminous and eccentric (Figure 1D). Follicles with two or more layers of cuboidal granulosa cells without antral cavity were classified as secondary follicles (Figure 1E–G). Early secondary follicles lacked zona pellucida (ZP), whereas the oocyte and granulosa cells appeared juxtaposed (Figure 1E). While the secondary follicle increased in numbers of granulosa cell layers apposed, the ZP was formed and became thicker and thicker (Figure 1F–H). The oocytes of tertiary follicles had a cumulus–oocyte complex (COC) where the oocyte was surrounded by corona radiata linked to mural granulosa cells by a mound of cumulus oophorous and floating in the antrum. The thecal cells wrapped the mural granulosa cells and the two follicular cell masses were separated by the basement membrane. At this stage, all oocytes were surrounded by a thick ZP layer (Figure 1I).

### 3.2. Ultrastructures of Follicles Examined by TEM

#### 3.2.1. Primordial Follicles

**Figure 2:**
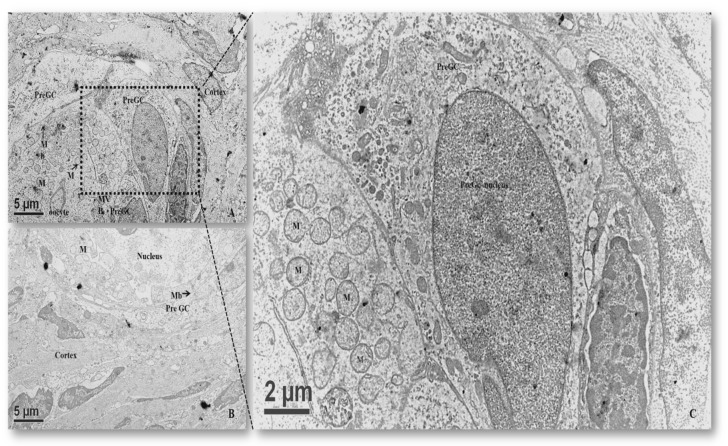
Transmission electron microscopic images of porcine primordial follicles showing the ultrastructure of oocytes and the associated cellular components: (**A**) a primordial follicle having juxtaposed flattened pregranulosa cells; (**B**) a primordial follicle with a voluminous eccentric oocyte nucleus (encompassed dot line); and (**C**) oocytes from the primordial follicles containing round and oval mitochondria with tiny cristae and electrodense matrices. Gc, granulosa cell; M, mitochondria; MV, mitochondria–vesicles complexes; Mb, cytoplasmic membrane.

In primordial follicles, each oocyte was embodied by a single layer of squamous epithelial cells known as pregranulosa cells (Figure 2A). Cell membranes of the oocyte and adjacent granulosa cells were juxtapositionally aligned. These cells were flattened and their nuclei were mostly elongated and crenellated shaped (Figure 2A). Occasionally, two or more clusters of primordial follicles were observed and their oolemma with some tight junctions. The oocyte nucleus occupied either a central or eccentric position of the ooplasm (Figure 2), and mitochondria were oval or roundish with electro-dense matrices (Figure 2C). Mitochondria with tiny cristae at a pole were sparsely distributed in the ooplasm (Figure 2C). Mitochondria aggregated into a complex associated with vesicles and unknown electro-dense components forming mitochondria–vesicles (MV) complexes, located close to the nucleus (Figure 2C).

#### 3.2.2. Primary Follicles

**Figure 3:**
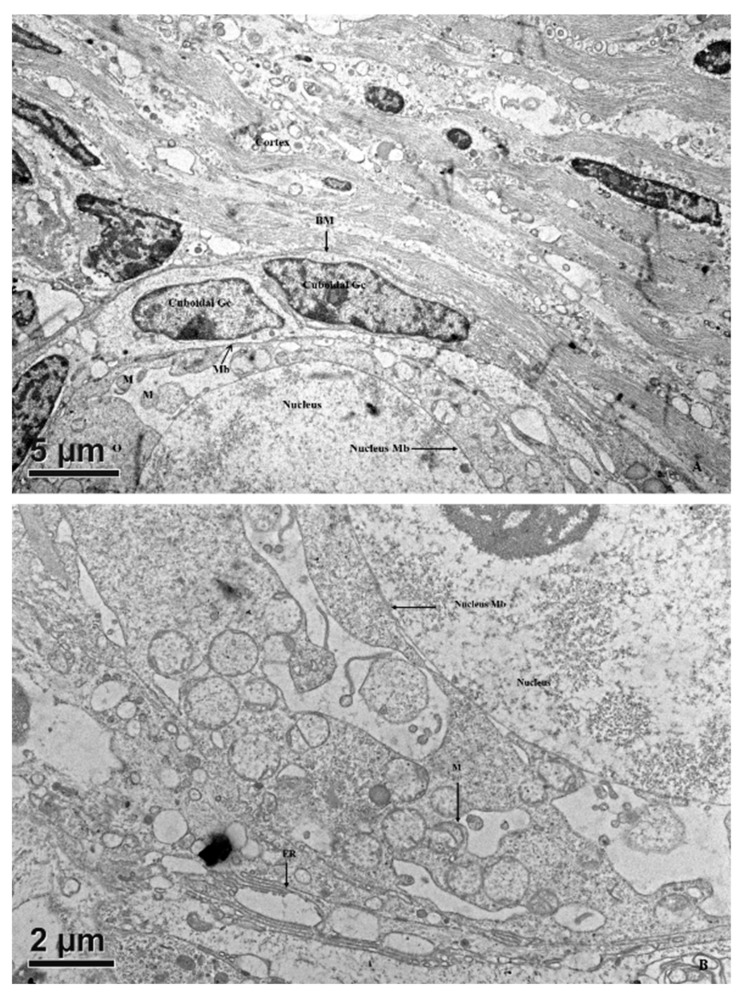
Transmission electron microscopic images showing the ultrastructure of oocytes and their surrounding cells in porcine primary follicles. (**A**) An early primary follicle with one layer of cuboidal granulosa cells beneath the basement membrane (BM) possessing an eccentric nucleus and clusters of organelles. (**B**) Note the clustering of organelles consists of mitochondria, endoplasmic reticulum, and vesicles, which are all tightened by the cement-like structure. The primary follicle has an even distribution of organelles throughout the ooplasm. Granulosa cells (GCs) are cuboidal in shape and oocyte cytoplasm contains mitochondria (M). ER, endoplasmic reticulum; O, oocyte; N, nucleus; M, mitochondrion; Mb, membrane.

The predominant spherical oocyte was surrounded by a single layer of granulosa cells (Figure 3). The oocytes of primary follicles were spherical or oval, with an initial eccentric nucleus when all of the granulosa cells were not yet cuboidal. Mitochondria were round and had electro-dense matrices. Oocytes from late primordial follicles enclosed by cuboidal granulosa cells had their organelles evenly distributed throughout the ooplasm. The ooplasm was full of vesicles, which appeared to form coalescent structures more frequently than that of the early primordial follicles. At this stage, the surrounding granulosa cells became cuboidal, and endoplasmic reticulum were rarely observed in the ooplasm; their cytoplasmic ultrastructure was similar to that of the primordial follicles.

#### 3.2.3. Secondary Follicles

**Figure 4:**
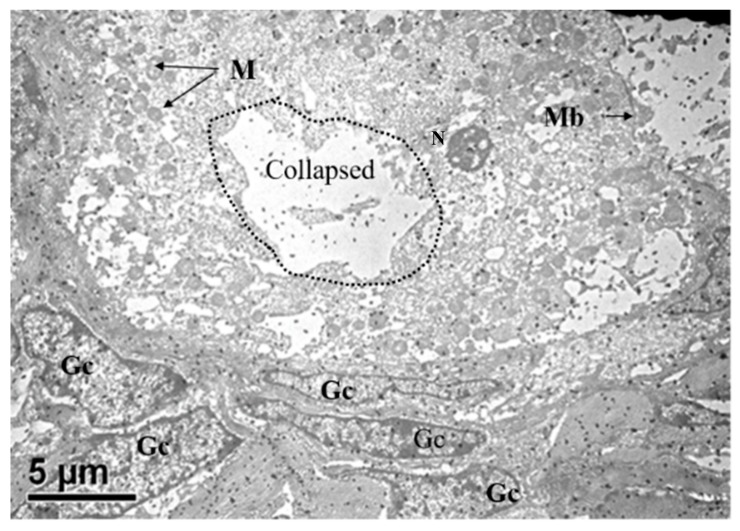
Transmission electron microscopy showing ultrastructure of oocytes (O) enclosed by multilayers of granulosa cells (GCs) in the secondary follicle. The secondary follicle reveals evenly distributed mitochondria. N, nucleus.

In the secondary follicle, oocytes were surrounded by more than one complete layer of cuboidal granulosa cells. Early secondary follicles had not yet displayed a well-developed zona pellucida. Large coalescent vesicles (collapsed) were observed compromising the visualization of organelles (Figure 4). In the secondary follicle, oocytes with dense organelles were mostly located at the periphery of the ooplasm. Mitochondrial density increased and their matrices were greyish in color. Their morphology was mostly round or oval with increased size compared to that of primordial and primary follicles. 

#### 3.2.4. Tertiary Follicles

**Figure 5:**
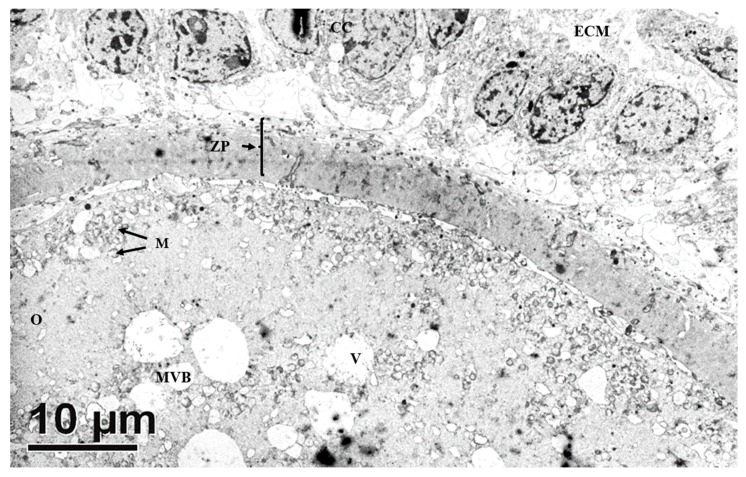
Transmission electron microscopic view of immature oocytes from antral follicles. The oocyte has fully formed zona pellucida (ZP), compact cumulus cells with minimal extracellular matrix (ECM), organelle aggregates, and clusters of mitochondria (M) beneath the vitelline membrane and in the ooplasm. CC, cumulus cell; M, mitochondrion; O, oocyte.

**Figure 6:**
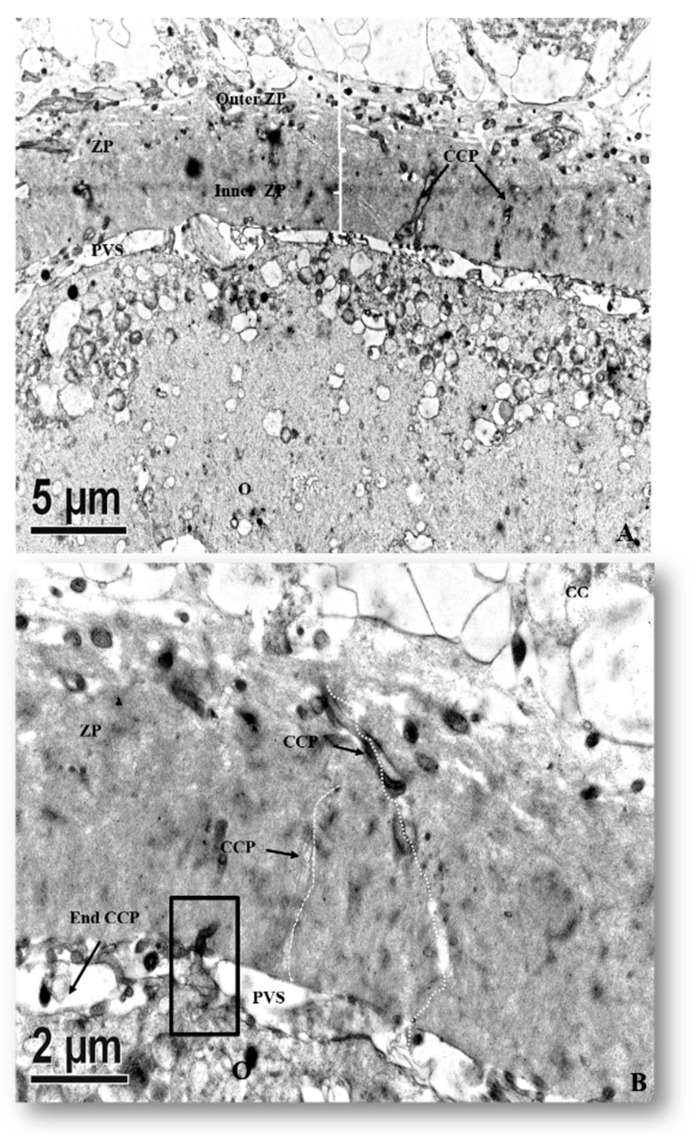
Ultrastructure of the transzona protrusion (TZP) from cumulus cells through the zona pellucida (ZP) to the oolemma membrane. (**A**) Ultrastructural details of cumulus–oocyte complexes showing the ZP is traversed by the cumulus cell projection (CCP) or TZP into the perivitelline space (PVS). (**B**) Zona pellucida of an immature oocyte presenting some CCPs that either end in the PVS (arrow) or form a tight junction (rectangle box) with the plasma membrane (Mb) of oocytes.

**Figure 7:**
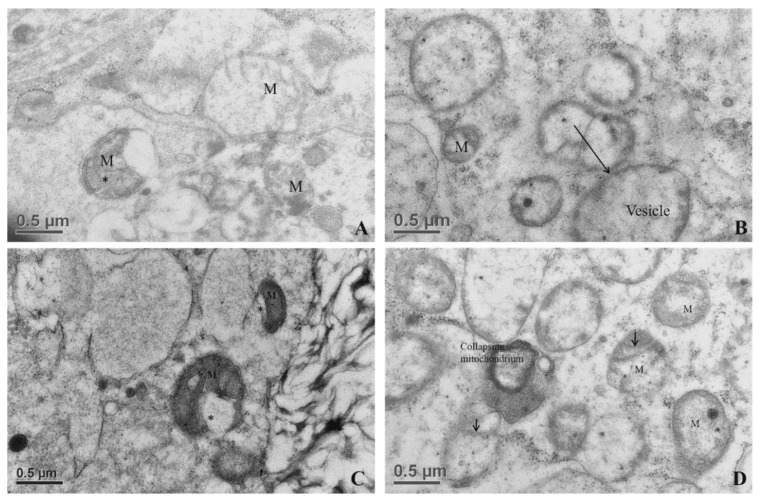
Ultrastructures of the organelles in oocytes of tertiary follicles. (**A**) Ooplasmic aggregate of organelles in the oocyte. (**B**) Aggregates of vesicles containing electron-dense component in association with mitochondria (mitochondria–vesicle (MV) complexes). (**C**) Electron micrograph of an immature oocyte with hooded mitochondria (*) in the aggregate of organelles. Aggregates of mitochondria associated with vesicles and lipid droplets. In the immature ooplasm, lipid droplets have less electron-dense and granule-like structures. Hooded mitochondrion matrices possessing well-developed cristae (arrow). (**D**) Collapsing mitochondrion was observed in the tertiary follicle oocyte. M, mitochondrion.

All tertiary follicles contained a spherical oocyte surrounded by the ZP and enclosed by tightly packed granulosa cells with much reduced extracellular matrix (ECM) (Figure 5). At this stage, granulosa cells surrounding the oocyte are now termed cumulus cells (CCs). The most inner granulosa cells closely associated with the zona pellucida named corona radiata, possessed projections through the ZP that ended at the indentations on the oolemma (rectangle box, Figure 6) or the perivitelline space (PVS). Zona pellucida had two zones: the outer ZP contained many cavities manifested as a sponge like-structure, while the inner zone is relatively firmer (Figure 6A). The perivitelline space was gradually reduced from which erected and bent microvilli of oolemma penetrated through the ZP (Figure 6B). Aggregated organelle structures (Figure 7A) consisting of mitochondria possessing developed cristae, vesicles, and other organelles bound with an electron-lucent structure (Figure 7A,B). Mitochondria with pleomorphic morphologies such as hooded (that appears in the section open to the cytoplasm, and like internal vesicles marked with * sign conformation were also observed (Figure 7A,C). Three oocytes out of eleven evaluated oocytes revealed presence of hooded mitochondria. The density of lipid droplets increased and were presented as a uniform and electro-dense streak-like structure (Figure 7C). Oocyte mitochondria and their complexes formed clusters in the ooplasm. These mitochondria were roundish and had darker matrices, as well as the most developed long cristae (Figure 7A,B,D). Collapsing mitochondrion was observed in tertiary follicle oocyte (Figure 7D).

#### 3.2.5. In Vitro Matured Oocytes

**Figure 8:**
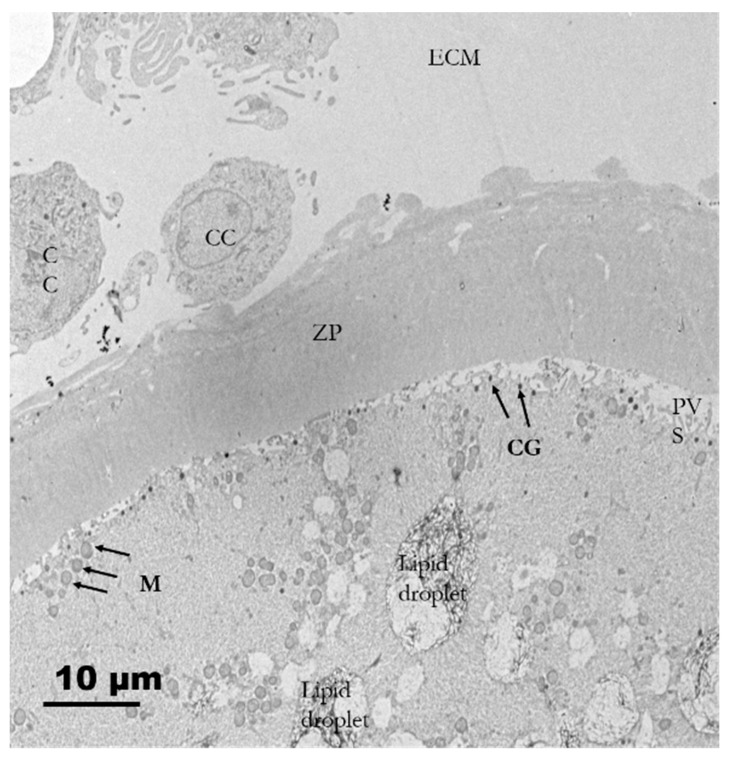
Electron micrograph of matured porcine oocytes (O). Note that the increased extracellular matrix (ECM) between cumuli cells (CCs) and the retracted cytoplasmic projections from cumulus cells against the oolemma are observed. Clusters of mitochondria are distributed in the ooplasm. A single ring of cortical granules (CGs) is present beneath the plasma membrane.

**Figure 9:**
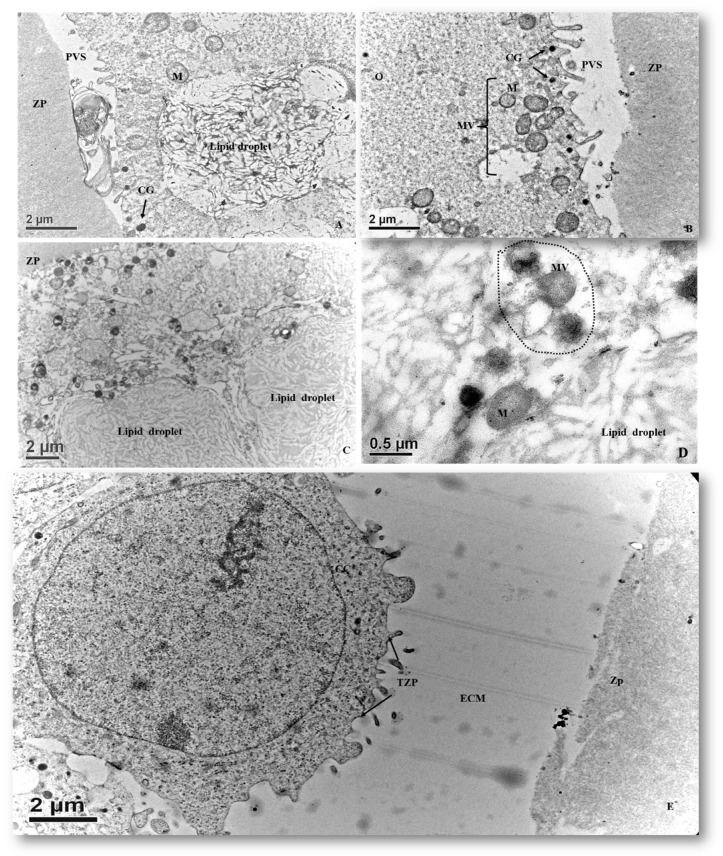
Ultrastructures of a matured porcine oocyte. (**A**) Electron micrograph of a matured oocyte with extruded polar body (PB) in the perivitelline space (PVS). (**B**) An electron micrograph of a matured oocyte. Note that the ruptures of cytoplasmic projections are visible in the PVS. The cortical granules (CGs) are more electron-dense (black) and form a ring-like structure beneath the oolemma membranes. (**C**) An electron micrograph showing lipid droplets in a matured oocyte. These lipid droplets are multi-structured and possess an electron-dense and streak-like morphology with mixed spots or border that are more grayish and amorphous. (**D**) Mitochondria are roundish with electrodense matrices that are centrifugally located in association with other organelles. (**E**) A micrograph of cumulus cells surrounding a matured oocyte. Note the enlarged ECM and retracted cytoplasmic projections, i.e., TZP. M, mitochondria; ZP, zona pellucida.

Matured oocytes enclosed in the cumulus–oocyte complexes (COCs) were characterized by an increased ECM volume among the surrounding cumulus cells. Cumulus cells were loosely compacted and initially had a lamellipodia-like projection toward the oocyte and increased perivitelline space (PVS). Clusters of mitochondria were distributed in the ooplasm. Cortical granules (CGs) appeared during the maturation process and were positioned beneath the oolemma, forming a single-layered ring-like structure (Figure 8). The first polar body (PB) was extruded into the PVS in which relatively darker and flocculent material was observed. PB was surrounded by several residual cytoplasmic droplets (Figure 9A). The most prominent organelles in a matured pig oocyte were some membrane-bound vesicles (large vesicles), lipid droplets and mitochondria (Figure 9B). The size of lipid droplets increased after maturation and their appearance varied from uniform electron-lucent streak to multiform streak-like structures, having different electro-dense grey spots within the droplets or on the border of vitelline membranes (Figure 9C). At this stage, the morphology of mitochondria was roundish or oval possessing electro-dense matrices. The mitochondria also bound to ERs (mostly smooth ERs) and lipid droplets form MVBs (Figure 9D). We also observed disruption of transzona protrusions (TZPs) throughout the ZP and the retracted TZPs toward cumulus cells (Figure 9E).

#### 3.2.6. Mitochondrial Morphometry

**Figure 10:**
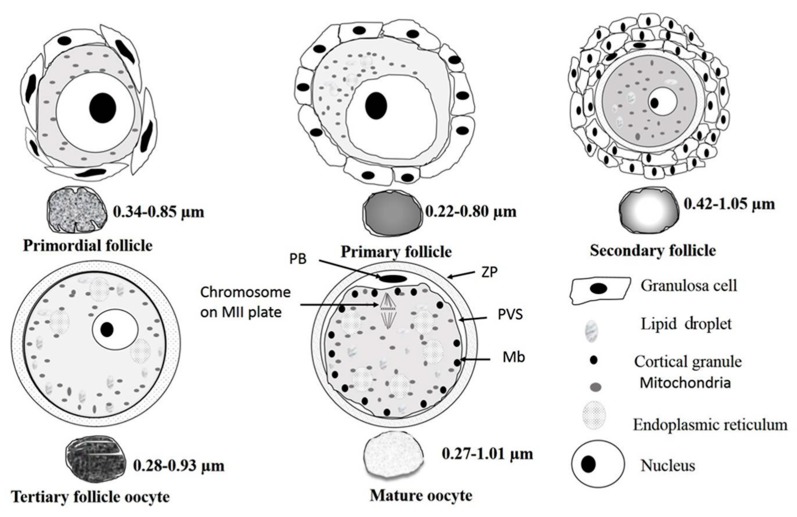
A summary diagram to illustrate mitochondrial morphodynamics in porcine oocytes during folliculogenesis toward meiotic maturation. Microtopography, size, shape, configuration of cristae, and matric density of the mitochondria change along the course of folliculogenesis. Oocyte mitochondria of the primordial follicle are roundish and the matrix is granite-like, and that of oocytes from primary follicles have darker matrices. The shape of mitochondria in the oocyte of secondary follicles is more elongated with vacuolated matrices. Oocytes of tertiary follicle also possess round mitochondria with elongated cristae and darker matrices. Mitochondria of in vitro matured oocytes have evenly distributed grayish matrices. Chronological and topomorphological changes of some major aggregates and organelle re-arrangement are observable during folliculogenesis and oocyte maturation.

The overall morphology of oocytes from different developmental stages of follicles is summarized in Figure 10. The mitochondrial morphometric values of oocytes derived from primordial, primary, secondary, and tertiary follicles, as well as that of in vitro matured oocytes, are presented in Table 1. Mitochondria of oocytes from primary and secondary follicles had apparently different diameters (*p* < 0.05). 

## 4. Discussion

We aimed to investigate the ultrastructural changes of porcine oocytes during folliculogenesis and in vitro maturation using electron microscope. Morphological characteristics of primordial, primary, secondary, and tertiary follicles are depicted in Figure 1A. In the present study, the observed primordial follicles with squamous (flattened) GCs and a few cuboidal GCs at one pole (Figure 1B) were the activated primordial follicle, as suggested by Fair et al. [18]. They are also named as the intermediary follicle because of the possession of both flattened and cuboidal unilaminar granulosa cells [18]. Oocytes from primordial follicles had only a few mitochondria sparsely distributed in the ooplasm. A similar pattern was described in human oocytes during the leptotene phase of meiosis, and was considered as a transitional stage because mitochondria only occupied the perinuclear position at the zygotene [1,31,32]. Oocytes of primordial and primary follicles contained mainly roundish or oval mitochondria, in which matrices were less electron-dense. Our findings are in agreement with those of previous investigations where pig oocytes of the primordial follicle possessed mitochondria that were spherical or egg-shaped with light matrix [17,18]. In growing follicles, mitochondria of the oocyte increased in number and also dispersed in the ooplasm [25,33]. Among organelles, we found that roundish mitochondria were the most abundant one in porcine oocytes of all follicular stages. Also, the mitochondrion diameter of pig oocytes decreased from primordial to primary follicle. Oocyte mitochondria of the secondary follicles were larger and were mostly similar in size in matured oocytes, while mitochondrion diameters from the tertiary follicle oocytes was relatively smaller (Table 1). The ooplasmic distribution of mitochondria in oocyte from secondary follicles differed from that repported by Silva et al. [18], who obtained a string of pearl organization of mitochondria. Compared to that of human’s, the size of pig mitochondria has evolved to become much smaller. Briefly, in humans, mitochondrial dimension increases from the dividing oogonia to the oocytes of primordial and primary follicles, reaching a diameter of 1–1.5 µm, but later a slight reduction (0.5–0.7 µm) along the course of folliculogenesis was observed [1,32]. 

Surprisingly, few hooded mitochondria were found in the tertiary follicle oocytes (3/11 oocyte). The hooded mitochondria were similar to those found in cattle [31,33,34,35]. Unlike ruminant (sheep and cattle) oocytes and early embryos [31,33,34,35,36,37,38], formation of hooded mitochondria was infrequently observed in porcine oocytes matured in vitro [16]. We infer that the hooded mitochondria found in our study might denote a response to a suboptimal growing environment. Immature oocytes had their mitochondria and their relative aggregates located beneath oolemma, whereas they formed an even distribution in the ooplasm of mature oocytes, in line with the findings of Sun et al. [37]. In the present study, the ZP surrounding oocytes appeared in the secondary follicles (Figure 1F). It differed from oocytes of others species which had ZP apposition starting as early as the primary follicle stage in guinea pigs [38], rabbits [39], humans [40,41], mice [42], cats [43], and dogs [44,45]. Our study revealed two structurally different layers of ZPs. The outer layer, much closer to cumulus cells, was a sponge-like structure presenting many cavities (Figure 7A). These cavities diminished in size from the outer to the inner part of the ZP; therefore, the inner ZP became a firm and continuous layer (Figure 7A). These findings are in agreement with those reported by Suzuki et al. [46], observed using scanning microscopy. Furthermore, granulosa cell projections proceeded through ZP and ended either in the perivitelline space or at the tight junctions with the oolemma (Figure 7B). Evidence indicates that somatic cell–oocyte interactions via gap junctions are essential for oocyte growth and metabolisms. They are also a critical portal for transportation of ions, nucleotides, amino acids, ATP, and pyruvate to the oocyte [12,15,47].

From secondary follicle oocytes to matured oocytes, numerous electron-lucent structures with various conformation and sizes are found to be lipid droplets in the present study ( Figure 5, Figure 7A,C,D, Figure 8, and Figure 9A,C). Throughout this study, we noticed the presence of bright vesicles in the ooplasm; however, we could not accurately define them due to the absence of appropriate staining. Therefore, some lipid droplets might have been misclassified as vesicles in other previous studies. In addition, the morphological changes in lipid droplets during folliculogenesis concurred with the changes in the nature of the lipids stored in those droplets. Isachenko and coworkers [48] suggested that the observed changes resulted from cytoplasmic lipolysis, i.e.: dark vesicles changing to gray ones after lipid utilization. In this study, intermediate patterns of the lipid droplets were frequently observed.

In the oocytes, mitochondrial-smooth ER aggregates (M-SER) and the mitochondrial-vesicle complexes (MV) are known to be involved in producing a reservoir of substances or membranes to participate in fertilization and early embryogenesis [1]. In the present study, cortical granules were mainly confined to the cortical region of the ooplasm throughout maturation and formed a ring-like structure. Matured oocytes had their connections via cumulus cell processes to oolemma membrane disrupted gradually, and the increase of PVS paralleled with the extrusion of the first PB, which was found as a flocculent structure in the present study. Similar observations were also reported in bovine and in vitro matured mouse oocytes [49,50].

## 5. Conclusions

Oocyte mitochondria changed in morphology, populated in different domains of the ooplasm, and established complicate connections with other organelles during meiotic maturation. Mitochondrial diameters and appearance also change significantly during the folliculogenesis and after in vitro maturation. This is the first report describing hooded mitochondria in growing porcine oocytes from the tertiary follicles. Moreover, alterations in the appearance of lipid droplets in growing oocytes implicate changes in the nature or status of lipid metabolisms, but more studies are required to bridge the physiologic gap between the organelles and oocyte developmental competency.

## Figures and Tables

**Table 1:** Mitochondrion diameters (µm) of oocytes from various stages of folliculogenesis and after in vitro maturation.

Follicular/Oocytes Stages	Primordial Follicle	Primary Follicle	Secondary Follicle	Tertiary Follicle	Matured Oocyte
Mean ± SEM	0.60 ± 0.03 ^ab^	0.51 ± 0.03 ^a^	0.73 ± 0.03 ^b^	0.59 ± 0.03 ^ab^	0.69 ± 0.03 ^ab^
Minimum	0.340	0.223	0.416	0.276	0.272
Maximum	0.859	0.803	1.047	0.926	1.014
No. of mitochondria measured	28	28	33	29	31
No. of oocytes evaluated by TEM	9	11	7	11	12
No. of oocytes used for mitochondria evaluation	5	5	4	4	4
No. oocyte hooded mitochondria	0	0	0	3	0

^a,b^ Means without the same superscript differed; No, Number.
